# Spinopelvic balance: new biomechanical insights with clinical implications for Parkinson’s Disease

**DOI:** 10.1186/1748-7161-9-S1-O41

**Published:** 2014-12-04

**Authors:** Luciano Bissolotti, Massimiliano Gobbo, Jorge Hugo Villafane, Stefano Negrini

**Affiliations:** 1 Fondazione Teresa Camplani Casa di Cura Domus Salutis, Brescia, Italy; 2 Department of Clinical and Experimental Sciences, University of Brescia, Brescia, Italy; 3 IRCCS Fondazione Don Gnocchi ONLUS, Milan, Italy; 4 Physical and Rehabilitation Medicine, University of Brescia, Italy, IRCCS Fondazione Don Gnocchi ONLUS, Milan, Italy

## Background

Compared to the amount of data regarding non neurogenic deformities, scientific papers focusing on sacro-pelvic morphology and sagittal balance in patients affected by Parkinson's Disease (PD) are represented by few case studies or cohorts.

## Aim

The aim of this study was to describe the disease-related sagittal balance changes in relation to the sacropelvic morphology of PD patients with different duration of disease.

## Methods

Thirty-one consecutive PD patients (26 males, 5 females; mean age 69.5±7.9years, range 55-83 years) participated in the cross sectional study. The clinical assessment included: Hohen Yahr score; plumb line distance from C7, kyphosis apex, L3 and S1. Lumbar lordosis, thoracic kyphosis, spinosacral angle, spinopelvic angle, spinal tilt, pelvic incidence, sacral slope and pelvic tilt were radiographically assessed.

## Results

Radiographic spinopelvic angles appeared normal, but many patients presented variations from normality. In particular, pelvic tilt increased (21.2±12.1°) and sacral slope decreased (33.8±10.9°); spinosacral (108.8±20.2°) and spinopelvic angles (150.3±17.1°) were reduced compared to healthy people. Five patients (16.1%) were presenting a camptocormic behavior while walking; 21 patients (67.7%) had an anterior decompensation (average PKA 32.6±27.7mm) while 10 (32.3%) had a posterior one (average distance PS1 24.5±9.4mm); 8 patients (25.8%) had a scoliosis above 20° (Cobb angle=30.8±14.4°), while 11 (35.4%) presented a mild form between 11° and 20° (Cobb angle= 11.2±2.1°). Even if the average value of radiographic parameters appeared normal (Table 2), 3 patients (9.6%) had a LL below 20°, and 3 (9.6%) a lumbar kyphosis; 12 (38.7%) presented a TK above 55°.

## Conclusions

Sagittal balance evaluation provides new valuable insights for biomechanical understanding of PD patients. Specific spinal parameters (spinosacral, spinopelvic and spinal tilt angles), and their clinical correlation, as well as pelvic parameters like pelvic tilt and sacral slope, appear particularly interesting for their clinical implications in terms of spinal deformities correction in PD population.

